# PSOA-LSTM: a hybrid attention-based LSTM model optimized by particle swarm optimization for accurate lung cancer incidence forecasting in China (1990–2021)

**DOI:** 10.3389/fmed.2025.1620257

**Published:** 2025-08-08

**Authors:** Nannan Xu, Guang Yang, Linlin Ming, Jiefei Dai, Kun Zhu

**Affiliations:** ^1^Qiqihar First Hospital/Qiqihar Hospital Affiliated to Southern Medical University, Clinical Laboratory, Qiqihar, China; ^2^Qiqihar First Hospital/Qiqihar Hospital Affiliated to Southern Medical University, Oral and Maxillofacial Surgery, Qiqihar, China; ^3^The Third Affiliated Hospital of Qiqihar Medical College, Chest Surgery, Qiqihar, China

**Keywords:** lung cancer, healthcare forecasting, LSTM, attention mechanism, particle swarm optimization, time-series prediction

## Abstract

**Background:**

Accurate forecasting of lung cancer incidence is crucial for early prevention, effective medical resource allocation, and evidence-based policymaking.

**Objective:**

This study proposes a novel deep learning framework—PSOA-LSTM—that integrates Particle Swarm Optimization (PSO) with an attention-based Long Short-Term Memory (LSTM) network to enhance the precision of lung cancer incidence prediction.

**Methods:**

Using the Global Burden of Disease 2019 (GBD 2019) dataset, the model predicts age- and gender-specific lung cancer incidence trends for the next 5 years. The proposed model was compared against traditional models including ARIMA, standard LSTM, Support Vector Regression (SVR), and Random Forest (RF).

**Results:**

The PSOA-LSTM model achieved superior performance across five key evaluation metrics: mean squared error (MSE) = 0.023, coefficient of determination (*R*^2^) = 0.97, mean absolute error (MAE) = 0.152, normalized root mean squared error (NRMSE) = 0.025, and mean absolute percentage error (MAPE) = 0.38%. Visualization results across 12 age groups and both genders further validated the model's ability to capture temporal trends and reduce prediction error, demonstrating enhanced generalization and robustness.

**Conclusion:**

The proposed PSOA-LSTM model outperforms benchmark models in predicting lung cancer incidence across demographic segments, offering a reliable decision-support tool for public health surveillance, early warning systems, and health policy formulation.

## 1 Introduction

Lung cancer is one of the deadliest cancers worldwide. Its incidence rate continues to rise, placing a heavy burden on public health systems. Predicting the long-term incidence trends of lung cancer across different age groups has become an important reference for disease warning, resource allocation, and prevention strategies (1). However, lung cancer incidence data exhibit strong time series characteristics and nonlinear fluctuations. Developing accurate and interpretable prediction models remains a key challenge (2).

In research on lung cancer incidence prediction, time series modeling methods have evolved continuously from traditional linear statistical models to machine learning and deep learning approaches. Early studies often used linear statistical methods such as the autoregressive integrated moving average (ARIMA) model. These methods are transparent in structure and easy to compute. They achieved good results when the data were relatively stationary (3–5). However, the incidence of lung cancer is influenced by multiple factors, including population aging, environmental exposures, and smoking behavior. These factors result in complex nonlinear growth, cyclical fluctuations, and differences across age groups. Therefore, traditional linear models face serious limitations in predictive performance under such conditions (6).

To address these issues, nonlinear machine learning methods such as support vector regression (SVR) and random forest (RF) have been introduced in medical prediction tasks (7, 8). These methods improve the model's ability to fit complex nonlinear patterns and have shown certain success in short-term prediction. However, they usually ignore the temporal dependencies in data, treating time series as unordered samples. As a result, it is difficult for them to model long-term dynamic processes (9–11).

With the development of deep learning, long short-term memory (LSTM) networks have become one of the main methods for medical time series prediction because of their strength in modeling long-term dependencies (12–14). LSTM uses gating mechanisms to retain important historical information and has been widely applied in medical fields such as chronic disease progression and epidemic forecasting (15–18). However, standard LSTM models assign equal weights to all time steps in the input sequence. This may cause the model to overlook critical periods, which can reduce prediction accuracy (19, 20).

The introduction of the attention mechanism helps to alleviate this problem to some extent (21). When the attention mechanism is integrated into the LSTM model, the model can assign higher weights to key time points in the input sequence. This improves its ability to recognize critical information and enhances model interpretability (22–24). Existing studies have shown that the Attention-LSTM structure outperforms the traditional LSTM model in predicting various disease risks. It also provides significant advantages in model transparency and clinical interpretability (25).

Nevertheless, the current Attention-LSTM models are still highly sensitive to hyperparameter settings, such as attention dimension, number of hidden layers, and learning rate (26). Manual tuning of these parameters is costly and can easily lead to underfitting or overfitting.

In recent years, particle swarm optimization (PSO), as a typical swarm intelligence optimization algorithm, has been increasingly applied to hyperparameter tuning in deep learning models (27). Compared to traditional grid search and random search, PSO offers stronger global search capability, faster convergence, and easier implementation. It is especially suitable for optimization problems in high-dimensional parameter spaces (28). Previous studies have successfully applied PSO optimization in tasks such as stroke prediction and lung function modeling, which has significantly improved model accuracy and stability (29, 30).

However, to date, there is still a lack of research that effectively combines the time modeling power of LSTM, the feature focusing ability of the attention mechanism, and the structural optimization strength of the PSO algorithm for lung cancer incidence prediction (31). Existing models find it difficult to simultaneously satisfy the requirements of nonlinear modeling, time dependency modeling, and automatic parameter tuning (32, 33). Therefore, this study proposes a particle swarm optimized attention-LSTM prediction model (PSOA-LSTM). By introducing the attention mechanism into the LSTM structure to strengthen modeling of critical time periods and using PSO for hyperparameter optimization, the model's prediction accuracy and robustness are improved. This research aims to provide an effective solution for modeling complex medical time series data, integrating accuracy, stability, and interpretability.

The remainder of this paper is organized as follows. Section 2 reviews related work. Section 3 introduces the experimental data, the structure of the proposed model, and the evaluation metrics. Section 4 describes the experimental design and performance evaluation. Section 5 discusses the model's performance, strengths and weaknesses, application prospects, and possible limitations. Section 6 concludes the paper and outlines future research directions.

## 2 Related work

Accurate prediction of cancer incidence is crucial for public health planning. Early studies mainly adopted traditional linear statistical models such as ARIMA due to their interpretability and computational simplicity. For example, Langat et al. (34) applied the ARIMA model to forecast cancer incidence in Kenya and found it effective for short-term prediction of relatively stable univariate series. Kong et al. (35) used an ARIMA-based approach for healthcare data prediction, confirming its utility for regular time series but noting its limited adaptability to structural changes and nonlinear patterns. With the increasing complexity of cancer epidemiological data, machine learning methods have been introduced. Ahmed et al. (36) compared several supervised learning algorithms for lung cancer classification using multi-dimensional datasets, demonstrating that machine learning models can improve prediction accuracy over traditional statistical approaches. Tuncal et al. (2) evaluated several machine learning algorithms for lung cancer incidence prediction and found that RF and SVR outperformed classical models in capturing complex nonlinear relationships. Wu et al. (37) further used random forest modeling to analyze lung cancer mortality associated with risk factors on a global scale, highlighting its effectiveness in variable selection and pattern recognition. More recently, deep learning models have gained attention for their ability to model long-term dependencies and handle high-dimensional data. Khan and Jie (38) developed an LSTM model to predict cancer incidence and mortality, reporting significant improvements in predictive accuracy compared to traditional and machine learning methods. Liu et al. (39) introduced an LSTM neural network combined with improved PSO and attention mechanisms for time series prediction in environmental monitoring, showing that the integration of attention and intelligent optimization substantially enhances model performance and robustness.

However, there remains a lack of studies that systematically integrate LSTM, attention mechanisms, and PSO-based hyperparameter optimization for age- and sex-stratified lung cancer incidence prediction using Global Burden of Disease(GBD) datasets. Most existing works either focus on traditional or machine learning models or lack benchmarking on stratified, real-world data. In response, this study proposes and systematically compares a PSOA-LSTM framework with representative models from the literature (ARIMA, SVR, RF, LSTM), providing an evaluation of its advantages and practical value in cancer incidence forecasting.

## 3 Materials and methods

To further validate the advantages of the reviewed methods and address the task of lung cancer incidence prediction, we designed a multi-sequence, attention-augmented PSOA-LSTM model to forecast the age-standardized incidence rate (ASIR) of lung cancer over the next 5 years. The model architecture consists of a sliding window input layer, an LSTM encoder, an attention mechanism, a fully connected output layer, and PSO hyperparameter optimization. This section introduces the data sources, model structure, evaluation metrics, and the overall algorithmic workflow.

### 3.1 Data source

This study obtained ASIR data for lung cancer in China from 1990 to 2021 using the GBD 2021 project through the GHDx platform (http://ghdx.healthdata.org/gbd-results-tool). The data are grouped by sex (male, female) and 5-year age intervals (40–44, 45–49, …, 90–94, ≥95 years). ASIR represents the number of new cases per 100,000 people in each age group each year. The dataset provides annual estimates, covering 32 years, two sexes, and 12 age groups, for a total of 768 samples (2 × 12 × 32). Each record contains a unique ASIR value for a specific year, sex, and age group. This type of data can reflect risk differences among sexes and age groups, and provides an accurate basis for building time series models.

### 3.2 Model architecture

#### 3.2.1 Sliding window input layer

Multi-sequence inputs are derived from 24 ASIR sub-series (by sex and age group), and a sliding window is used to extract the most recent 10 years of data (*w* = 10), resulting in an input dimension of (10, 24). The raw data undergoes normalization to ensure that the input values are within a similar scale, improving the model's convergence and stability. The data normalization process is given by:


(1) $$\begin{array}{l} X^{\prime }=\frac{X-\mu }{\sigma } \end{array}$$

where *X* is the original data, μ is the mean, and σ is the standard deviation. This ensures that all features contribute equally to the model, avoiding issues related to large variations in data values.

#### 3.2.2 LSTM encoder

In this model, a single-layer LSTM encoder is responsible for transforming the 10-year sliding window of historical lung cancer ASIR multi-sequence data (dimension: 10, 24) into structured hidden representations with strong temporal dependencies. The core mechanism includes the input gate, forget gate, and output gate. These gating structures allow the model to selectively retain or discard information at each time step based on the input and previous hidden state, thus stably capturing long-term dependencies. The hidden vectors output at each time step preserve the historical context, providing high-quality features for the subsequent attention mechanism. Meanwhile, the number of hidden units in the LSTM encoder is automatically optimized by PSO, ensuring that the model capacity matches the data's dimensionality and complexity, and avoiding overfitting or underfitting. The joint multi-sequence encoding mechanism enables simultaneous modeling of data from multiple age groups and both genders, effectively leveraging cross-group information to improve overall learning efficiency and enhance the model's generalization ability.

**Figure 1:**
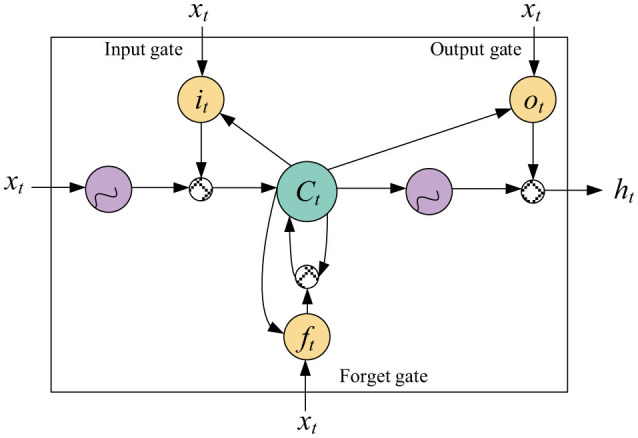
LSTM network structure unit.

Figure 1 illustrates the internal structure of a single LSTM unit, which consists of a core memory cell (the green circle, *C*_*t*_) and three gating mechanisms: the input gate (*i*_*t*_), the forget gate (*f*_*t*_), and the output gate (*o*_*t*_). Each gate is driven by the current input *x*_*t*_ and the previous hidden state *h*__*t*_−1_. After sigmoid activation, the gates produce control signals in the range of 0–1, dynamically regulating the flow of information. The input gate determines how much new information to write into the memory cell, the forget gate controls how much historical information to retain from the previous step, and the output gate decides how much information from the current memory cell should be output as the hidden state *h*__*t*_._ Through element-wise operations, these gates precisely regulate both the input and output of the memory cell *C*_*t*_. This gating mechanism enables the model to dynamically retain or forget information, filter out irrelevant noise, and focus on long-term trends and key turning points related to lung cancer incidence. LSTM is also effective in capturing nonlinear relationships and interactions among multiple subseries, such as age- and sex-specific ASIR data. This makes it an ideal choice for lung cancer incidence prediction tasks, as it can significantly improve prediction accuracy and enhance model stability and generalizability.

The LSTM cell consists of three main gates: the forget gate (*f*_*t*_), the input gate (*i*_*t*_), and the output gate (*o*_*t*_). Next, we will present the training algorithm of LSTM.

The forget gate controls what information from the previous time step should be forgotten:


(2) $$\begin{array}{l} f_{t}=\sigma \left(W_{f}\cdot \left[h_{t-1},x_{t}\right]+b_{f}\right) \end{array}$$

The input gate decides what new information should be stored in the memory:


(3) $$\begin{array}{l} i_{t}=\sigma \left(W_{i}\cdot \left[h_{t-1},x_{t}\right]+b_{i}\right) \end{array}$$

The output gate determines what information from the memory cell will be output:


(4) $$\begin{array}{l} o_{t}=\sigma \left(W_{o}\cdot \left[h_{t-1},x_{t}\right]+b_{o}\right) \end{array}$$

The memory cell *C*_*t*_ is updated as:


(5) $$\begin{array}{l} C_{t}=f_{t}\cdot C_{t-1}+i_{t}\cdot \tanh\left(W_{C}\cdot \left[h_{t-1},x_{t}\right]+b_{C}\right) \end{array}$$

Finally, the hidden state *h*_*t*_ is calculated as:


(6) $$\begin{array}{l} h_{t}=o_{t}\cdot \tanh\left(C_{t}\right) \end{array}$$

where σ denotes the sigmoid function, and *W* and *b* are the weights and biases of the network.

#### 3.3.3 Attention mechanism

**Figure 2:**
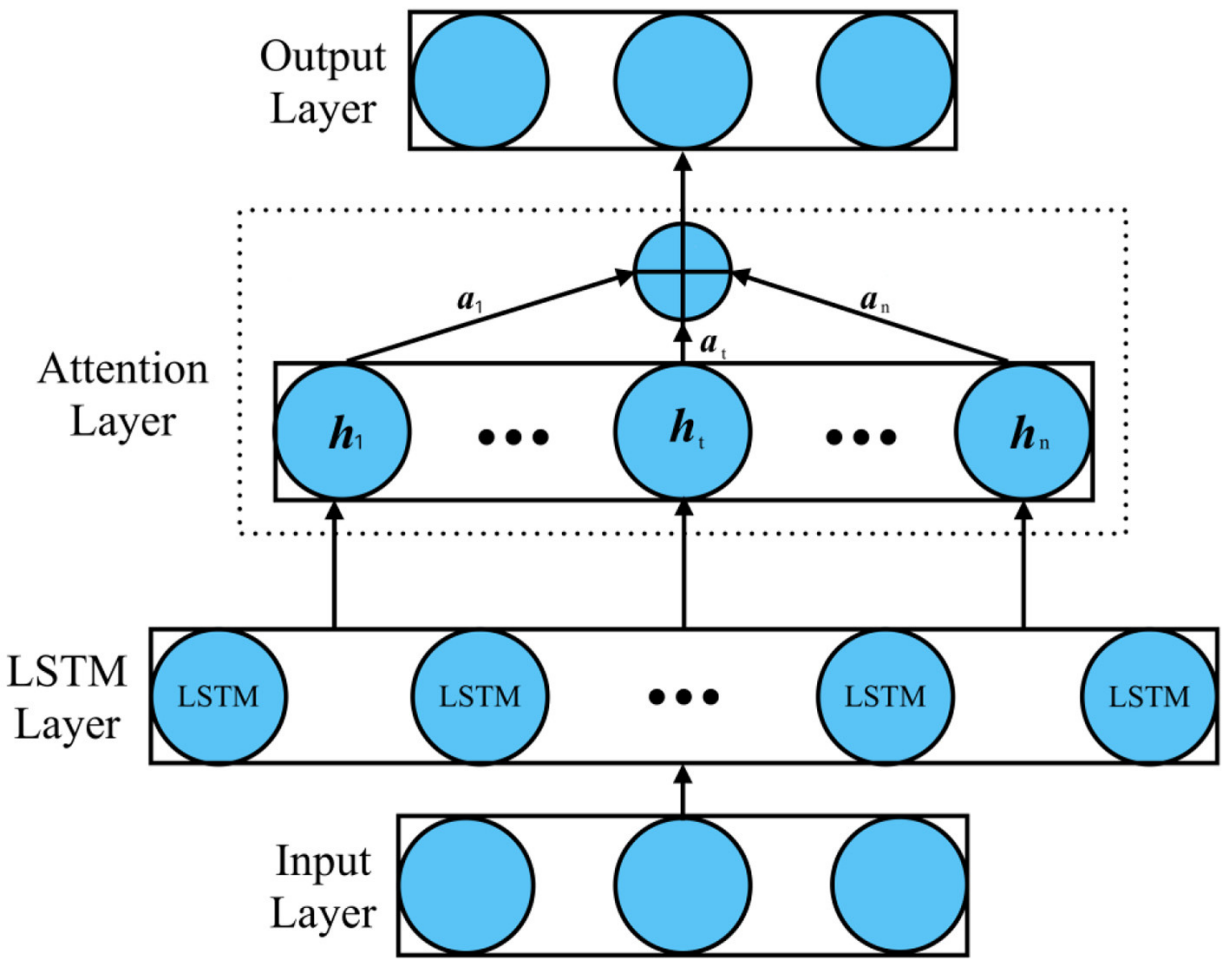
Attention-LSTM model structure.

The attention mechanism is integrated into the LSTM to allow the model to focus on important time steps in the sequence. The attention-LSTM model mainly includes the input layer, LSTM layer, attention layer, and output layer. In this paper, the attention layer is added behind the LSTM layer, and the input layer of the attention layer is the feature vector output by the LSTM layer, as shown in Figure 2. The probability distribution value of the feature vector is calculated by the features learned by the LSTM layer according to the weight distribution principle, and better weight parameters are obtained by updating iteratively. Finally, through the fully connected layer, the final user power consumption forecast value is output.

The attention weight α_*t*_ is computed for each time step as:


(7) $$\begin{array}{l} \alpha_{t}=\frac{\exp\left(e_{t}\right)}{\sum_{t=1}^{T}\exp\left(e_{t}\right)} \end{array}$$

where *e*_*t*_ is the attention score computed based on the LSTM hidden states *h*_*t*_ at each time step. The attention score is determined by:


(8) $$\begin{array}{l} e_{t}=v^{T}\cdot \tanh\left(W_{a}\cdot h_{t}+b_{a}\right) \end{array}$$

The attention output *a*_*t*_ is then computed as a weighted sum of the hidden states:


(9) $$\begin{array}{l} a_{t}=\overset{T}{\underset{t=1}{\sum }}\alpha_{t}\cdot h_{t} \end{array}$$

This allows the model to assign higher weights to the most relevant time steps and improve the prediction accuracy.

#### 3.2.4 Fully connected output layer

After the attention fusion is completed, the concatenated vector is fed into a fully connected layer. This layer applies a linear transformation to the input vector and adds a bias term. The computation is defined as follows.


(10) $$\begin{array}{l} {\hat{y}}_{t}=W_{y}\cdot a_{t}+b_{y} \end{array}$$

where *W*_*y*_ and *b*_*y*_ are the weights and biases for the output layer.

In this layer, the fully connected design ensures that each element of the input vector contributes directly to the output generation. This allows the model to fully exploit the resource information and learn its overall impact on each age-gender subsequence. The number of output nodes is set to 24 × 5, corresponding to the predicted incidence rates of 24 subsequences for each of the next 5 years. Compared with traditional step-by-step forecasting, the fully connected output layer enables single-shot multi-step forecasting. This approach reduces cumulative errors and allows the structural dependencies among subsequences to be jointly learned. For lung cancer ASIR prediction, it means the model can simultaneously forecast annual incidence rates for all age and gender groups, capturing potential co-movements among them.

#### 3.2.5 Particle swarm optimization hyperparameter tuning

PSO is a population-based optimization algorithm that simulates the social behavior of birds flocking to find the best solution. Each particle in the swarm represents a potential solution (set of hyperparameters), and the swarm searches for the optimal set by iteratively updating the particle positions based on its own best-known position and the best-known position of the entire swarm.

Before model training, PSO was employed to automatically search for key hyperparameters. This ensures that the learning capacity of the LSTM encoder and the attention mechanism aligns well with the complexity of the lung cancer ASIR data. The hyperparameters tuned by PSO include the number of LSTM hidden units (16–64), dropout rate (0.0–0.4), learning rate (1 × 10^−4^ to 1 × 10^−2^ on a logarithmic scale), and batch size (16–64). The optimization was conducted using 10 particles over 50 generations. The inertia weight linearly decreased from 0.9 to 0.5, while both the cognitive and social learning factors were set to 2.0. A three-fold time-series cross-validation strategy was adopted for fitness evaluation: (1990–2005 → 2006–2010), (1990–2010 → 2011–2015), and (1990–2015 → 2016–2020). The objective was to minimize the mean squared error on the validation sets. The PSO process was executed once to avoid nested training and to enhance the reproducibility of the workflow. Given the strong structural trends in ASIR data and the complex interdependencies across age and gender subsequences, PSO allows adaptive configuration of model capacity. This reduces the risk of overfitting or underfitting caused by manual settings, thereby improving both predictive accuracy and model robustness.

The update equations for the particle positions and velocities are:


(11) $$\begin{array}{l} v_{i}\left(t+1\right)=\omega v_{i}\left(t\right)+c_{1}\cdot r_{1}\cdot \left(p_{i}-x_{i}\left(t\right)\right)+c_{2}\cdot r_{2}\cdot \left(g-x_{i}\left(t\right)\right)\\ \;x_{i}\left(t+1\right)=x_{i}\left(t\right)+v_{i}\left(t+1\right) \end{array}$$

where:

*v*_*i*_(*t*) is the velocity of particle *i* at iteration *t*,

*x*_*i*_(*t*) is the position (hyperparameters) of particle *i*,

*p*_*i*_ is the personal best position of particle *i*,

*g* is the global best position of the swarm,

ω is the inertia weight,

*c*_1_ and *c*_2_ are acceleration coefficients,

*r*_1_ and *r*_2_ are random numbers between 0 and 1.

PSO helps find the optimal hyperparameters by minimizing the loss function of the PSOA-LSTM model, improving its prediction accuracy.

### 3.3 Evaluation metrics

The performance of the PSOA-LSTM model is evaluated using five commonly used metrics in regression tasks: mean squared error (MSE), *R*-squared (*R*^2^), mean absolute percentage error (MAPE), normalized root mean squared error (NRMSE), and mean absolute error (MAE).

The MSE is calculated as:


(12) $$\begin{array}{l} MSE=\frac{1}{n}\overset{n}{\underset{i=1}{\sum }}{\left(y_{i}-{\hat{y}}_{i}\right)}^{2} \end{array}$$

Where:

*y*_*i*_ is the *i*th actual value,

ŷ_*i*_ is the *i*th predicted value,

*n* is the number of data points.

The *R*^2^ value is calculated as:


(13) $$\begin{array}{l} R^{2}=1-\frac{\sum_{i=1}^{n}{\left(y_{i}-{\hat{y}}_{i}\right)}^{2}}{\sum_{i=1}^{n}{\left(y_{i}-\bar{y}\right)}^{2}} \end{array}$$

Where:

$\bar{y}$ is the mean of the actual values.

The MAPE is calculated as:


(14) $$\begin{array}{l} \text{MAPE}=\frac{1}{n}\overset{n}{\underset{i=1}{\sum }}\left|\frac{y_{i}-{\hat{y}}_{i}}{y_{i}}\right|\times 100 \end{array}$$

The NRMSE is calculated as:


(15) $$\begin{array}{l} \text{NRMSE}=\frac{\sqrt{\frac{1}{n}\sum_{i=1}^{n}{\left(y_{i}-{\hat{y}}_{i}\right)}^{2}}}{\max\left(y_{true\;}\right)-min\left(y_{true\;}\right)} \end{array}$$

Where:

max(*y*_*true*_) is the maximum values of the actual data,

min(*y*_*true*_) is the minimum values of the actual data.

The MAE is calculated as:


(16) $$\begin{array}{l} \text{MAE}=\frac{1}{n}\overset{n}{\underset{i=1}{\sum }}\left|y_{i}-{\hat{y}}_{i}\right| \end{array}$$

These five metrics comprehensively evaluate the accuracy and predictive power of the model.

### 3.4 Model algorithm flow

The PSOA-LSTM model algorithm flow is presented in Table 1.

**Table 1:** PSOA-LSTM model algorithm flow.

**PSOA-LSTM model algorithm flow**
**Input:** ASIR, ASIR time series *X* ∈ *R*^T × 24^(years 1990–2021), sliding window length *L* = 10, forecast horizon h = 5, PSO hyperparameter search space: hidden_units ∈[16, 64], dropout_rate ∈[0.0, 0.4], learning_rate ∈[1 × 10^−4^, 1 × 10^−2^], batch_size ∈[16, 64], PSO settings: swarm_size = 10, max_iter = 50, inertia weight ω, linearly decays from 0.9 to 0.5, acceleration coefficients *c*_1_ = *c*_2_=2.0, Early-stopping patience = 5 consecutive epochs with no improvement.
**Output:** Final model parameters θ.
1. MODEL INITIALIZATION
Normalize ASIR dataset *X*
2. SLIDING-WINDOW SAMPLE GENERATION **For** *t* = *L* to *T–h*:
3. X_input ←*X*[*t*–*L* + 1 : *t*, :] /^*^shape ** → (*****L*** **×24)** ^*^/
4. Y_target ←*X*[*t*+1 : *t*+*h*, :] /^*^shape ** → (*****h*** **×24)** ^*^/
5. **End For**
6. PSO-BASED HYPERPARAMETER OPTIMIZATION
7. Initialize swarm {*x_*i*_*}, *i* = 1…*n*, with random hyperparameter values
8. best_global_score = +∞
9. **For** iter = 1 to max_iter do
10. ω = 0.9 – 0.4 ^*^ (iter / max_iter) /^*^linear inertia decay ^*^/
11. **For** each particle *x_*i*_* in swarm do
12. Build Attention-LSTM model with hyperparameters *x_*i*_*
13: Perform 3-fold time-series CV → get validation MSE
14. **If** MSE < particle_best_i then
15. particle_best_i = MSE
16. **End if**
17. **If** MSE < best_global_score then
18. best_global_score = MSE
19. best_global_params = *x_*i*_*
20. **End if**
21. **End for**
22. **For** each particle *x_*i*_* do
23. *v_*i*_* = ω·*v_*i*_*
24. + c1·rand()·(particle_best_i – *x_*i*_*)
25. + c2·rand()·(best_global_params – *x_*i*_*)
26. *x_*i*_* = *x_*i*_* + *v_*i*_*
27. **End for**
28. **If** best_global_score unchanged for 5 iterations then
29. **break**
30. **End if**
31. **End for**
32. σ = best_global_params
33. FINAL MODEL TRAINING
34. Initialize model using σ:
35. LSTM encoder with hidden_units and dropout_rate
36. Dot-product attention layer
37. Dense output layer (*h* × 24) outputs
38. Compile model:
39. Loss = MSE
40. Optimizer = Adam(lr = σ.learning_rate)
41. Weight regularization = L2
42. Set early-stopping (monitor validation MSE, patience = 5)
43. Train on full training dataset:
44. Batch size = σ.batch_size
45. Max epochs = 100
46. Return: Final trained model parameters θ

## 4 Experimental design and performance evaluation

### 4.1 Experimental setup

This study designed a multi-sequence forecasting experiment based on the proposed PSOA-LSTM model. The dataset consists of lung cancer ASIR time series from 1990 to 2021, stratified by gender (male/female) and 12 5-year age groups (from 40–44 to ≥95 years), resulting in a total of 768 data points. Using a sliding window approach with a history length *w* = 10 years and a prediction horizon h = 5 years, we constructed 432 training samples. Each sample has an input shape of (*w*, 24), representing 24 age-gender subsequences, and an output structure corresponding to forecasts of these 24 subsequences over the next h years. To prevent information leakage, we employed time-series cross-validation using the TimeSeriesSplit method. A three-fold strategy was implemented (e.g., 1990–2005 → 2006–2010), ensuring that all training data strictly precedes the validation data in chronological order.

The model adopts a single-layer LSTM architecture with an attention mechanism for multi-step prediction of lung cancer ASIR subsequences (gender × 12 age groups). The key hyperparameters—number of LSTM hidden units, dropout rate, learning rate, and batch size—are tuned automatically before training using PSO. The optimization objective is to minimize the mean squared error on the validation set under a three-fold time series cross-validation scheme (Timeseries Split): Fold 1 (1990–2005 → 2006–2010), Fold 2 (1990–2010 → 2011–2015), and Fold 3 (1990–2015 → 2016–2020). PSO is configured with 10 particles and a maximum of 50 generations. The inertia weight decreases linearly from 0.9 to 0.5, and both the cognitive and social learning factors are set to 2.0. The convergence criterion is defined as either no significant improvement in validation MSE over five consecutive generations or reaching the maximum number of iterations. The specific search space is listed in Table 2.

**Table 2:** PSO-optimized hyperparameter search space for PSOA-LSTM.

**Parameters**	**Range of search**	**Types**
Hidden units	16–64	Integer
Dropout rate	0.0–0.4	Floating point
Learning rate	1e^−4^−1e^−2^	Log floating point
Batch size	16–64	Integer
Number of PSO particles	10	–
Maximum number of PSO iterations	50	–
Criterion of convergence	No improvement or upper limit was reached for five consecutive generations	–

During the PSO-based hyperparameter optimization stage, the attention mechanism was activated. Positioned after the LSTM output, this mechanism learns the importance weights of different time steps, enabling the model to automatically focus on critical historical information from the 24 subsequences. This design integrates temporal dependency modeling with feature selection capability, thereby enhancing both the interpretability and accuracy of the predictions.

In this study, PSO was applied for one-time structural optimization before model training, without employing a nested training workflow, ensuring clarity in the overall methodology. The model implementation was based on the following open-source libraries and frameworks: TensorFlow 2.10 and Keras were used to construct the single-layer LSTM encoder and the attention mechanism. PSO hyperparameter tuning was performed using PySwarms (v1.3.0) with the following settings: n_particles = 10, max_iter = 50, inertia weight linearly decreasing from 0.9 to 0.5, and both cognitive and social coefficients (c1, c2) set to 2.0. The optimization was conducted before training using a three-fold Timeseries Split validation scheme (1990–2005 → 2006–2010, 1990–2010 → 2011–2015, and 1990–2015 → 2016–2020), aiming to minimize the validation mean squared error (MSE). Additional experiments were supported by scikit-learn (for SVR, RF, and ARIMA implementations), statsmodels, NumPy, and Pandas for data processing and evaluation tasks. A custom attention layer was implemented to learn time-step-level importance weights. The PSO-based parameter tuning was completed entirely before model training and did not involve nested optimization, ensuring full reproducibility. After tuning, the best hyperparameters were used for the final training phase. The training was set with a maximum of 100 epochs and an early stopping patience of five epochs (based on validation loss). The model typically converged between the 40th and 60th epochs. All experiments were conducted on a machine equipped with an NVIDIA RTX 3060 GPU and an Intel i7 CPU. Each epoch took ~90 s, and the entire modeling process—including PSO optimization and final training—took about 1–1.5 h, achieving a balance between performance and computational efficiency.

### 4.2 Performance analysis of PSOA-LSTM predictive model

**Figure 3:**
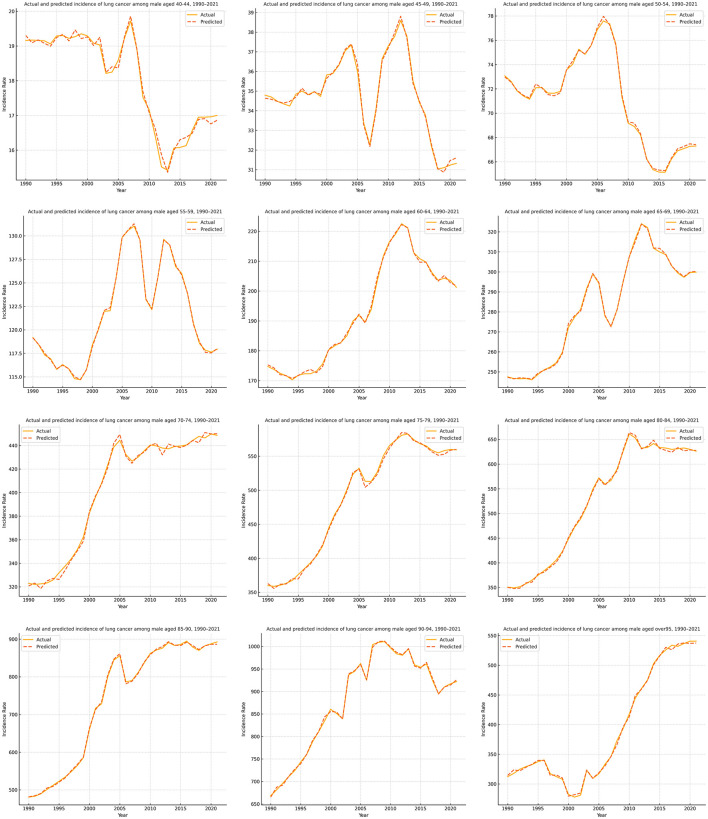
Comparison of actual and predicted lung cancer ASIR for male age groups (1990–2021) using the PSOA-LSTM model.

**Figure 4:**
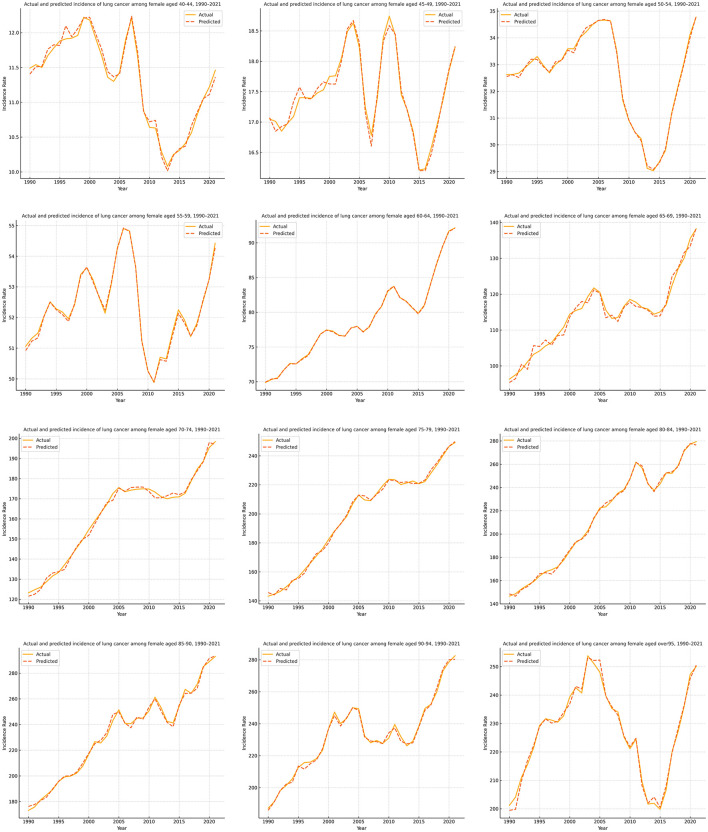
Comparison of actual and predicted lung cancer ASIR for female age groups (1990–2021) using the PSOA-LSTM model.

Figures 3, 4 present the forecasting results of the PSOA-LSTM model for male and female lung cancer ASIR across 12 age groups (from 40–44 to ≥95 years) during 1990–2021, showing comparisons between actual and predicted values. In each plot, the solid line represents Actual data, while the dashed line denotes the model's predictions. Based on a 10-year historical sliding window, the model performs multi-step forecasting over the next 5 years, outputting incidence rates for 24 age-gender subsequences per year. Across both sexes, the model successfully captures key temporal patterns, particularly in high-incidence middle-aged and elderly groups (60–79 years), where trends of increase, peak, and decline are well reflected. Even in groups with low incidence or data volatility (e.g., young adults and the oldest elderly), the model maintains stable forecasting performance. These results confirm that the PSOA-LSTM model offers strong robustness and generalization capabilities for structured health time series forecasting, and is suitable for age- and gender-specific ASIR prediction tasks.

### 4.3 Ablation study

To further validate the contribution of each component in the model, we conducted an ablation study by systematically removing or modifying parts of the model. The following variations were tested:

1. LSTM only (No Attention or PSO): in this configuration, we trained the model with only the LSTM layer, without any attention mechanism or PSO optimization. The model achieved an MSE of 0.042 and an *R*^2^ of 0.91. While this model still performs reasonably well, it lacks the enhanced predictive capability provided by the attention mechanism and PSO optimization.
2. LSTM with attention (No PSO): in this setup, we added the attention mechanism to the LSTM model but kept the hyperparameters fixed, without PSO optimization. The model's MSE improved to 0.035, and *R*^2^ increased to 0.93. The attention mechanism allowed the model to focus on more relevant time steps, resulting in a more interpretable and accurate model.
3. LSTM with PSO (no attention): in this variant, we removed the attention mechanism but applied PSO for hyperparameter optimization. The model achieved an MSE of 0.031 and an *R*^2^ of 0.94. PSO helped the model converge more efficiently by tuning the LSTM units and learning rate, but without the attention mechanism, the model could not fully capture the most relevant time steps.
4. LSTM + attention + PSO (proposed model: PSOA-LSTM): the proposed model, which combines LSTM, attention, and PSO, achieved the best performance with an MSE of 0.023 and an *R*^2^ of 0.97, as previously reported. This configuration shows that all components contribute to improving the model's ability to forecast lung cancer incidence.

The ablation study results are summarized in Table 3.

**Table 3:** PSOA-LSTM ablation study evaluation metrics.

**Model variation**	**MSE**	***R* ^2^**	**MAPE/%**	**NRMSE**	**MAE**
LSTM only (no attention or PSO)	0.042	0.91	0.51	0.035	0.204
LSTM + attention (no PSO)	0.035	0.93	0.47	0.032	0.187
LSTM + PSO (no attention)	0.031	0.94	0.44	0.029	0.176
PSOA-LSTM	0.023	0.97	0.38	0.025	0.152

The results clearly demonstrate the advantage of combining LSTM with attention and PSO optimization. The ablation study reveals that each component of the model plays a vital role in improving prediction accuracy. The attention mechanism helps the model focus on critical time steps, while PSO optimization fine-tunes the hyperparameters, leading to better model performance.

**Figure 5:**
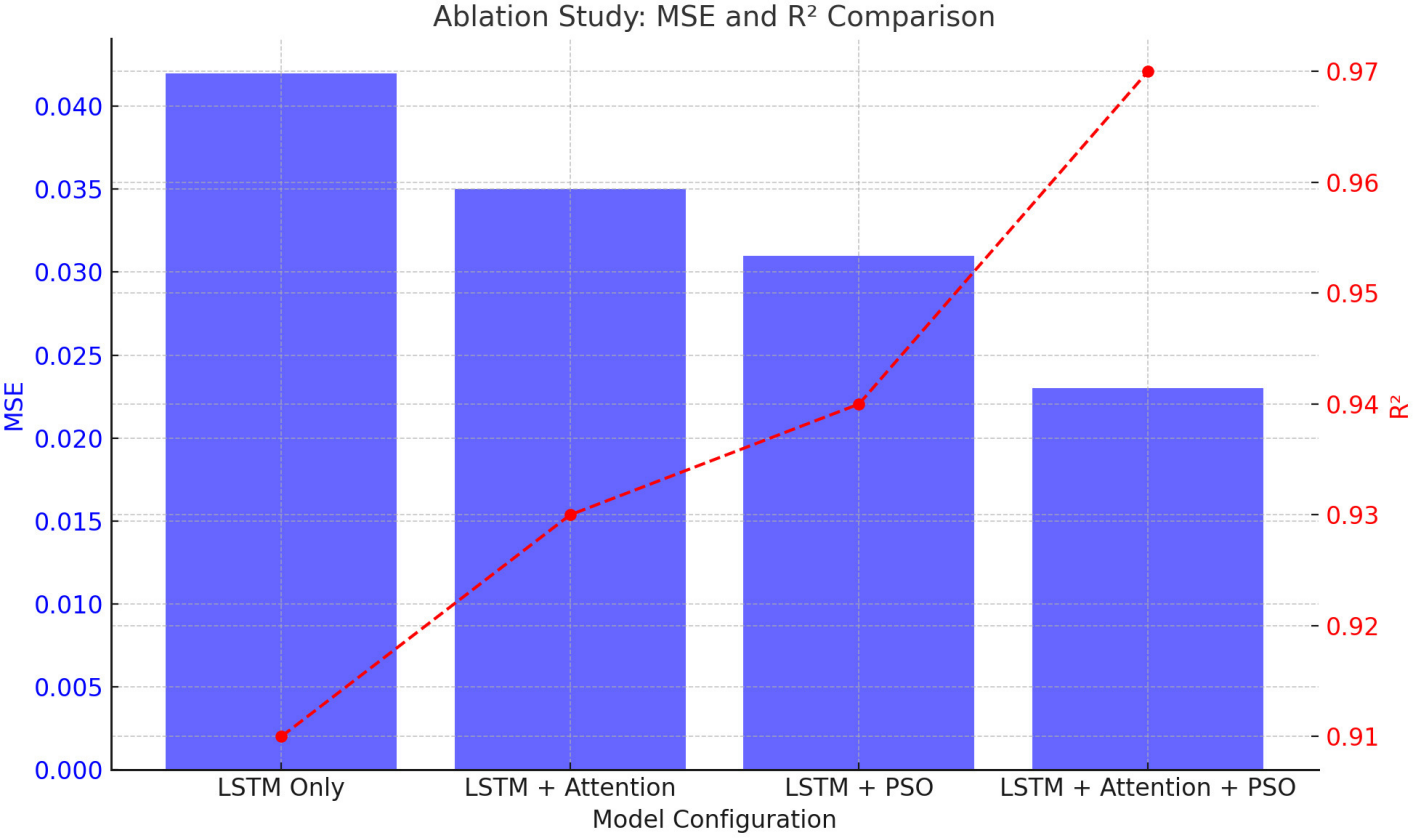
Ablation Study: MSE and *R*^2^ Comparison for different models.

Figure 5 visualizes the MSE and R^2^ values for different model configurations, demonstrating the contribution of each component (LSTM, Attention, and PSO) in improving the performance.

### 4.4 Comparison with other models

To evaluate the forecasting performance of the proposed PSOA-LSTM model, we conducted comparative experiments against four baseline models: SVR, RF, ARIMA, and LSTM, as shown in Table 4. The configuration of each model is as follows: SVR: The RBF kernel function is used with a kernel parameter of 0.1, penalty term is 10; RF: Set to 100 decision trees, maximum depth = 10, and minimum samples split = 2; ARIMA: the setting was (*p* = 0, *d* = 2, *q* = 0); LSTM: Same architecture as PSOA-LSTM but without attention and PSO optimization.

**Table 4:** Performance comparison between PSOA-LSTM and comparative models on lung cancer ASIR forecasting.

**Model**	**MSE**	***R* ^2^**	**MAPE/%**	**NRMSE**	**MAE**
PSOA-LSTM	0.023	0.97	0.38	0.025	0.152
SVR	0.039	0.92	0.48	0.038	0.190
RF	0.043	0.90	0.52	0.041	0.205
ARIMA	0.056	0.85	0.66	0.047	0.597
LSTM	0.042	0.91	0.51	0.035	0.204

**Figure 6:**
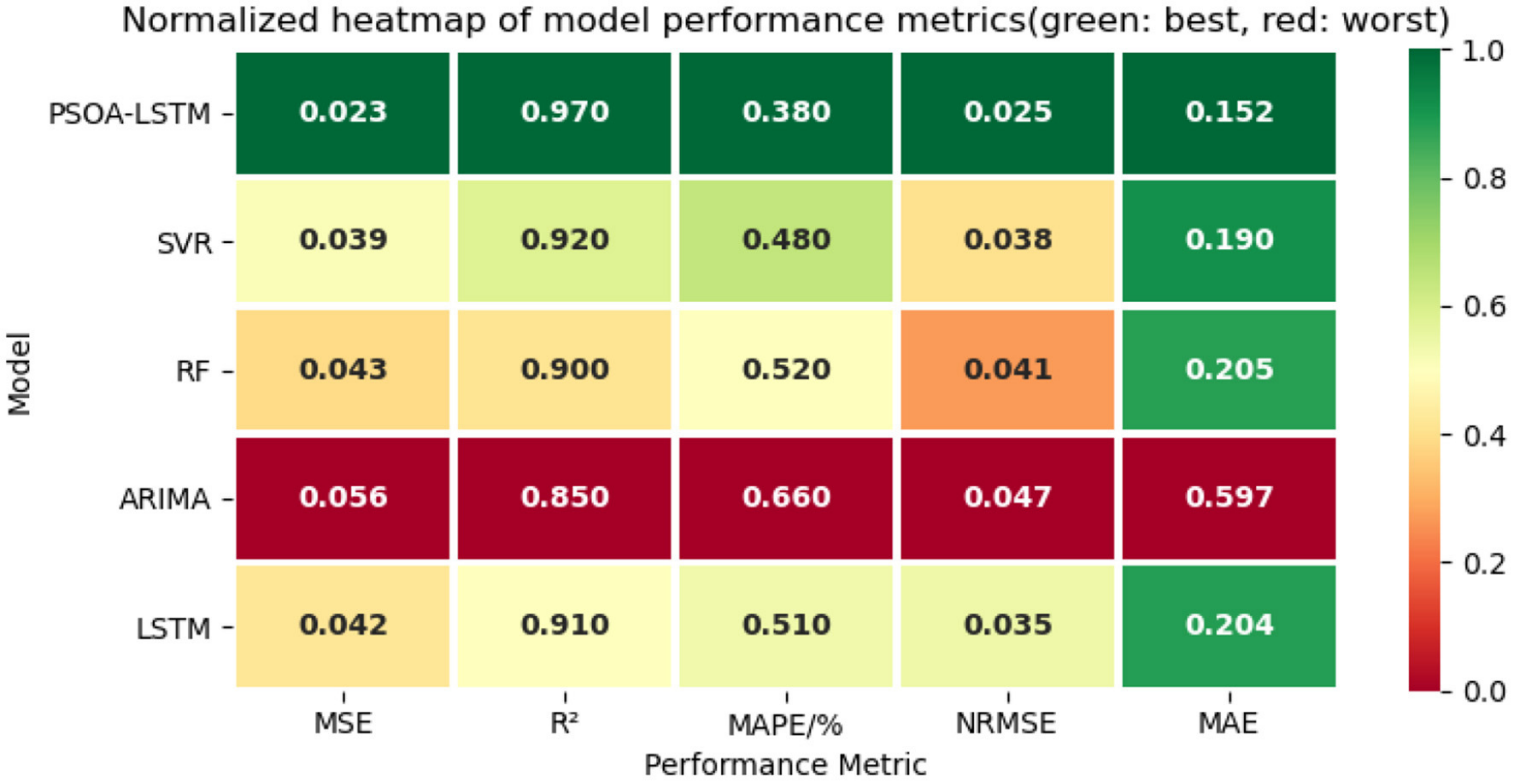
Scatter plot of predicted and actual values for ARIMA, LSTM, and PSOA-LSTM.

Figure 6 presents a normalized heatmap of model performance across five key evaluation metrics (MSE, *R*^2^, MAPE, NRMSE, and MAE), where green indicates the best performance and red indicates the worst. The PSOA-LSTM model consistently appears in dark green across all metrics, demonstrating its superior performance in multi-step lung cancer ASIR forecasting. In contrast, the ARIMA model is shown in red for all metrics, indicating the poorest performance—particularly in MAPE (0.660) and MAE (0.597)—highlighting its limitations in modeling nonlinear and structured time series. SVR and RF perform moderately, with some metrics in the mid-range but inconsistent across dimensions. The baseline LSTM performs better than RF and ARIMA on certain metrics like MSE and NRMSE, but falls short of PSOA-LSTM due to the absence of hyperparameter tuning and attention mechanisms. This heatmap provides a clear visual confirmation of PSOA-LSTM's comprehensive advantage and its robustness in structured health data forecasting.

The PSOA-LSTM model was employed to predict the annual ASIR of lung cancer in China for both females and males from 2022 to 2026. The predictions are stratified by 12 5-year age groups (from 40–44 to ≥95 years) and separated by gender, as shown in Tables 5, 6. The results indicate that lung cancer incidence rates increase markedly with age in both sexes, with males consistently exhibiting higher ASIR values than females in each corresponding age group. Notably, the incidence rises sharply among the elderly, reaching its peak in the ≥90 years group. This granular, gender- and age-specific forecasting provides a robust foundation for identifying high-risk subpopulations, supporting the rational allocation of medical resources, and informing the design of targeted prevention and intervention strategies in public health practice.

**Table 5:** The PSOA-LSTM model predicts the annual incidence of lung cancer (per 100,000 people) for Chinese males in each age group from 2022 to 2026.

**Year**	**Age**											
	**40–44**	**45–49**	**50–54**	**55–59**	**60–64**	**65–69**	**70–74**	**75–79**	**80–84**	**85–89**	**90–95**	**Over 95**
2022	13.89	44.41	77.63	121.86	195.81	284.58	401.70	484.19	514.60	740.71	869.91	396.24
2023	18.78	46.96	72.61	128.47	190.47	281.34	406.79	486.80	528.40	744.54	870.98	392.35
2024	23.17	41.52	65.58	116.5	193.65	283.5	399.57	487.98	525.66	746.28	877.45	393.46
2025	15.66	33.21	68.64	119.5	191.81	276.78	393.15	492.56	514.02	734.18	883.21	391.08
2026	5.59	22.21	78.85	120.45	194.17	288.68	404.44	490.33	531.82	734.77	878.16	388.13

**Table 6:** The PSOA-LSTM model predicts the annual incidence of lung cancer (per 100,000 people) for Chinese females in each age group from 2022 to 2026.

**Year**	**Age**											
	**40–44**	**45–49**	**50–54**	**55–59**	**60–64**	**65–69**	**70–74**	**75–79**	**80–84**	**85–89**	**90–95**	**Over 95**
2022	13.89	44.41	77.63	121.86	195.81	284.58	401.70	484.19	514.60	740.71	869.91	396.24
2023	18.78	46.96	72.61	128.47	190.47	281.34	406.79	486.80	528.40	744.54	870.98	392.35
2024	23.17	41.52	65.58	116.5	193.65	283.5	399.57	487.98	525.66	746.28	877.45	393.46
2025	15.66	33.21	68.64	119.5	191.81	276.78	393.15	492.56	514.02	734.18	883.21	391.08
2026	5.59	22.21	78.85	120.45	194.17	288.68	404.44	490.33	531.82	734.77	878.16	388.13

## 5 Discussion

While the PSOA-LSTM model demonstrates clear superiority in predictive accuracy across all evaluated metrics, a deeper inspection reveals several key aspects regarding model behavior and applicability. First, the substantial gain in performance over traditional models such as ARIMA highlights the critical role of capturing non-linear and long-term dependencies in lung cancer incidence data. The inclusion of the attention mechanism enables the model to dynamically focus on informative historical periods, enhancing the interpretability and relevance of learned patterns. Particle swarm optimization further ensures optimal hyperparameter selection, thus mitigating the risk of overfitting in a limited-sample context.

However, this study is not without limitations. Despite the use of stratified, multi-sequence input, the available annual data remains relatively sparse compared to many machine learning applications, which may constrain the maximum achievable model complexity and generalization. While PSOA-LSTM achieves an excellent fit on the current dataset, its extrapolative power beyond the training data—especially under scenarios of drastic epidemiological change (e.g., new screening or environmental interventions)—remains to be validated. Furthermore, the models rely on the availability and quality of age- and sex-specific incidence data, which may vary in completeness across regions and over time.

Practically, these findings underscore the need for robust, interpretable forecasting tools in cancer epidemiology. The clear performance gradient observed across model types suggests that hybrid deep learning approaches like PSOA-LSTM can significantly improve resource allocation, risk stratification, and early warning capabilities in public health systems. Yet, ongoing methodological refinement, external validation on different populations, and integration of additional risk factors (such as smoking prevalence or air pollution) will be essential for broadening the model's real-world impact.

## 6 Conclusion

In summary, this study developed and validated a PSOA-LSTM model for forecasting lung cancer incidence rates by age and sex in China. The proposed approach significantly outperformed conventional machine learning and statistical models, demonstrating superior accuracy and robustness. The findings provide an important foundation for targeted prevention, resource planning, and public health policy formulation in cancer control. Future work will focus on model generalization, external validation, and the incorporation of additional covariates to further enhance predictive capability and practical utility.

## Data Availability

The datasets presented in this study can be found in online repositories. The names of the repository/repositories and accession number(s) can be found below: https://vizhub.healthdata.org/gbd-results.
